# Probing the Interaction of Dielectric Nanoparticles with Supported Lipid Membrane Coatings on Nanoplasmonic Arrays

**DOI:** 10.3390/s17071484

**Published:** 2017-06-23

**Authors:** Abdul Rahim Ferhan, Gamaliel Junren Ma, Joshua A. Jackman, Tun Naw Sut, Jae Hyeon Park, Nam-Joon Cho

**Affiliations:** 1 School of Materials Science and Engineering and Centre for Biomimetic Sensor Science, Nanyang Technological University, 50 Nanyang Drive, Singapore 637553, Singapore; ferhan@ntu.edu.sg (A.R.F.); gma001@e.ntu.edu.sg (G.J.M.); jjackman@ntu.edu.sg (J.A.J.); suttun001@e.ntu.edu.sg (T.N.S.); park0005@e.ntu.edu.sg (J.H.P.); 2 School of Chemical and Biomedical Engineering, Nanyang Technological University, 62 Nanyang Drive, Singapore 637459, Singapore

**Keywords:** nanoplasmonics, localized surface plasmon resonance, biosensor, near field decay, supported lipid bilayer, membrane-nanoparticle interactions

## Abstract

The integration of supported lipid membranes with surface-based nanoplasmonic arrays provides a powerful sensing approach to investigate biointerfacial phenomena at membrane interfaces. While a growing number of lipid vesicles, protein, and nucleic acid systems have been explored with nanoplasmonic sensors, there has been only very limited investigation of the interactions between solution-phase nanomaterials and supported lipid membranes. Herein, we established a surface-based localized surface plasmon resonance (LSPR) sensing platform for probing the interaction of dielectric nanoparticles with supported lipid bilayer (SLB)-coated, plasmonic nanodisk arrays. A key emphasis was placed on controlling membrane functionality by tuning the membrane surface charge vis-à-vis lipid composition. The optical sensing properties of the bare and SLB-coated sensor surfaces were quantitatively compared, and provided an experimental approach to evaluate nanoparticle–membrane interactions across different SLB platforms. While the interaction of negatively-charged silica nanoparticles (SiNPs) with a zwitterionic SLB resulted in monotonic adsorption, a stronger interaction with a positively-charged SLB resulted in adsorption and lipid transfer from the SLB to the SiNP surface, in turn influencing the LSPR measurement responses based on the changing spatial proximity of transferred lipids relative to the sensor surface. Precoating SiNPs with bovine serum albumin (BSA) suppressed lipid transfer, resulting in monotonic adsorption onto both zwitterionic and positively-charged SLBs. Collectively, our findings contribute a quantitative understanding of how supported lipid membrane coatings influence the sensing performance of nanoplasmonic arrays, and demonstrate how the high surface sensitivity of nanoplasmonic sensors is well-suited for detecting the complex interactions between nanoparticles and lipid membranes.

## 1. Introduction

Ongoing advances in micro- and nanofabrication lend excellent potential for interfacing sensor technologies with biologically relevant components such as viruses [1,2,3], exosomes [4,5,6], and nanoparticles [7,8]. By fabricating sensor arrays on solid supports, a wide range of surface-sensitive measurement techniques can be utilized to investigate the binding and conformational properties of biomacromolecules and soft matter interacting with nanostructures. Within this scope, one of the most promising directions involves engineering nanostructured sensing arrays to probe interactions involving cell membranes [9,10,11]. Indeed, cell membranes are a key component of biological life, and membrane integrity is important for biological functions such as cellular compartmentalization and biochemical signaling [12,13]. From a design perspective, the topic is particularly interesting because supported lipid membrane fabrication is challenging and a vast number of functionalization possibilities exist by selecting the appropriate membrane composition. Conventionally, supported lipid membranes are formed on silica-coated surfaces, which promote spontaneous bilayer formation whereas the formation process is less favorable on other materials such as gold and titanium oxide [14].

To date, most studies involving supported lipid membrane fabrication and nanostructured arrays have been conducted using quartz crystal microbalance-dissipation (QCM-D) measurements [15]. A key motivation behind this thrust is that QCM-D has been widely used to study supported lipid membrane formation on flat surfaces and it is compatible with dielectric-coated sensing surfaces [16,17,18,19,20]. By contrast, other popular sensor techniques such as surface plasmon resonance (SPR) typically require metal-coated surfaces that are not compatible with membrane fabrication [21,22]. At the same time, fabricating nanostructures on QCM-D sensing platforms requires delicate attention to how nanostructure-associated hydrodynamic effects influence measurement responses [23,24,25]. Another promising measurement option involves utilizing nanoplasmonic sensors whereby the nanostructures not only contribute to the sensor geometry but also play an active role as the transducing element [26,27,28,29,30]. In particular, incident light can excite the conduction band of electrons in metal nanoparticles, resulting in a localized surface plasmon resonance (LSPR) that gives rise to a maximum intensity of light extinction at a certain wavelength (λ_max_) [31,32]. The resonance wavelength is highly sensitive to changes in the local dielectric environment and hence Δλ_max_ shifts can be monitored to detect binding events along with structural changes in biomacromolecular conformation [27]. For arrays of nanoparticles on a solid support, these measurements can be conducted at the single-particle level by measuring scattering intensity in a dark field illumination setup [33,34,35] or, at the ensemble-average level, by measuring changes in extinction intensity (absorption + elastic scattering) in transmission or reflection modes [36,37].

While LSPR-based measurements rely on the plasmonic properties of metal nanoparticles [32,38,39], there have been numerous advances in terms of surface functionalization that facilitate interfacing them with supported lipid membranes [40]. It is possible to coat gold or silver nanoparticles with self-assembled monolayers upon which a phospholipid monolayer can form [41,42]. Such capabilities have been utilized to form hybrid bilayers for detecting protein binding to membrane-embedded receptors such as biotin functional groups and carbohydrate moieties [43,44]. More recently, the concept of “indirect nanoplasmonic sensing” has become popular whereby the entire sensor surface is coated with a thin, conformal dielectric layer, oftentimes silica or titanium oxide [45]. This approach has broadly enabled the fabrication of fluidic, two-dimensional supported lipid bilayers (SLBs) on nanodisk [14,46,47,48,49] and nanohole [50,51,52] architectures, opening the door to a wide range of studies involving protein binding [11,47,53,54,55] as well as vesicle adsorption and deformation [56,57,58,59,60,61]. As these fabrication approaches gain traction, there has been renewed emphasis on varying the membrane composition to modulate SLB–substrate interactions (e.g., via membrane surface charge) as well as employing the nanostructured sensing platforms to study membrane interactions pertaining to various classes of molecules and nanomaterials such as amphipathic peptides [49], graphene oxide sheets [62], and ionic liquids [63]. Looking forward, one outstanding area of opportunity lies in achieving rational control over membrane interactions through appropriate surface functionalization, both within the context of fundamental biointerfacial science as well as developing model systems for biological systems.

The interactions between nanoparticles and lipid membranes are highly relevant to medicine, toxicology, and environmental science, and there is broad interest in utilizing label-free, surface-sensitive measurement techniques to characterize nanoparticle adsorption onto lipid membranes and accompanying changes in membrane properties [64,65,66,67]. Traditionally, changes in lipid membrane properties triggered by the interaction with specific biomacromolecules are studied in vitro on living cells using fluorescence imaging techniques [68,69]. While the adaptation of such techniques to study nanoparticle–membrane interactions enables qualitative analysis of cell uptake pathways and reveals eventual cell fate due to nanoparticle interactions, making them suitable for toxicity studies, they do not facilitate detailed quantitative analysis of the nanoparticle–membrane interaction and the monitoring of structural changes occurring at the nanoparticle–membrane interface due to interaction complexities arising from the large variety of involved components [70,71]. As such, surface-based model membrane systems have been developed to overcome these limitations [72,73,74]. In these surface-based systems, nanostructured surfaces such as nanodisks and nanoholes may be utilized as underlying substrates, which have consequently prompted studies on the interaction between nanostructured surfaces and lipid bilayers [75,76,77,78]. Well-characterized bilayer coatings have been employed to study lipid bilayer interactions with biomacromolecules [79,80,81] as well as with native [82,83,84] and functionalized nanoparticles [85,86,87,88,89] with consideration of various parameters such as nanoparticle size as well as surface property variations (e.g., charge, hydrophobicity) of both the nanoparticles and lipid bilayers. On a related note, some of these studies have been conducted using advanced label-free optical sensing techniques such as optical waveguide spectroscopy [80] and waveguide total internal reflection microscopy [85]. However, to date, nanoparticle–membrane interactions have not been studied by nanoplasmonic sensors.

Herein, we investigate the interaction between silica nanoparticles and SLB-coated gold nanodisk arrays, with particular emphasis on understanding how SLB properties can be modulated to influence nanoparticle–membrane interactions as well as characterizing how the SLB coating affects LSPR sensing properties. To approach this subject, we have selected spherical, 50-nm diameter silica nanoparticles as a model system and systematically investigated their interactions with SLBs of tunable composition possessing either neutral or positive membrane surface charges. As presented herein, our findings demonstrate that nanoplasmonic sensors are capable of not only measuring the rate of nanoparticle uptake but also detecting lipid composition-dependent interaction signatures arising from nanoparticle-induced SLB reconfigurations. The measurement capabilities were also utilized to probe the influence of precoating a synthetic protein corona on the silica nanoparticle surface, and clarified how the interplay of membrane surface charge and nanoparticle coating can be used to promote or inhibit nanoparticle–membrane interactions. All of these findings are discussed within the context of the high surface sensitivity of LSPR sensors and how this sensitivity is affected by SLB coatings, and further demonstrates the unique measurement capabilities of LSPR sensors to investigate dynamic processes occurring at lipid membrane interfaces.

## 2. Materials and Methods

### 2.1. Reagants

The phospholipids used for the preparation of vesicles, 1,2-dioleoyl-*sn*-glycero-3-phosphocholine (DOPC) and 1,2-dioleoyl-*sn*-glycero-3-ethylphosphocholine (chloride salt) (DOEPC) were purchased from Avanti Polar Lipids, Inc. (Alabaster, AL, USA). Silica nanoparticles dispersed in water were acquired from nanoComposix, Inc. (San Diego, CA, USA). Bovine serum albumin (BSA; A2153) was purchased from Sigma-Aldrich (St. Louis, MO, USA). All solutions were prepared in Milli-Q-treated water (>18 MΩ·cm) (Millipore, Billerica, MA, USA).

### 2.2. Dynamic Light Scattering

Dynamic light scattering (DLS) was used to determine the size distribution of lipid vesicles in bulk solution. Measurements were performed using a 90Plus particle size analyzer (Brookhaven Instruments, Holtsville, NY, USA) with a 658.0 nm monochromatic laser, and were taken at a 90° scattering angle to minimize reflection effects. The BIC Particle Sizing software package (Brookhaven Instruments) was used to measure and analyze the intensity autocorrelation function and to obtain the intensity-weighted Gaussian size distribution of the vesicles. The average effective diameter of all vesicles used was ~70 nm with a polydispersity of <0.1.

### 2.3. Vesicle Preparation

Phospholipid vesicles were prepared by the extrusion method. Lipids were mixed to the desired molar ratio in chloroform, and then dried with nitrogen gas to form a dried lipid film in a glass vial. In order to eliminate any residual chloroform, the dried lipid film was stored in a vacuum desiccator overnight. The lipid film was then hydrated with an aqueous buffer (10 mM Tris, 50 mM NaCl, pH 7.5) and vortexed for 3 min to form multilamellar vesicles. Subsequently, the vesicles were extruded through 50 nm-diameter polycarbonate membrane pores using a Mini-Extruder (Avanti Polar Lipids, Inc., Alabaster, AL, USA). The final stock concentration of lipid vesicles was 5 mg/mL, and vesicle dilution was performed in buffer solution immediately before experiment.

### 2.4. BSA Coating of Silica Nanoparticles

A 0.5 mL solution of silica nanoparticles (6 mg/mL) was mixed with a 0.5 mL solution of BSA (6 mg/mL) in aqueous buffer (10 mM Tris, 150 mM NaCl, pH 7). The mixture was incubated in a 1.5 mL Eppendorf centrifuge tube for 2 h at 37 °C in a thermal orbiter. The mixture was then centrifuged for 30 min at 16,000× *g* at room temperature. After centrifugation, 0.9 mL of the supernatant was discarded and replenished with fresh buffer before vortexing for 5 min. This washing step was repeated before the BSA-coated silica nanoparticles were immediately used.

### 2.5. Localized Surface Plasmon Resonance

LSPR experiments were conducted using the XNano instrument (Insplorion AB, Gothenburg, Sweden) that was operated in optical transmission mode, as previously described [60]. Briefly, ensemble-averaged measurements were obtained by monitoring the transmission of a white light beam (~4 mm^2^ circular spot) passing through transparent sensor chips that were obtained from Insplorion AB. The light beam subsequently exited through a quartz glass window and the light was then collected by a spectrophotometer. The sensor chips were made of fused silica containing well-separated and randomly distributed gold nanodisks on the surface (surface coverage ~8%), as produced by hole-mask lithography [90]. The sensor chip was sputter-coated with a silicon nitride film (thicknes ~10 nm), and each coated nanodisk had an average height and diameter of 20 and 120 nm, respectively, as characterized and reported in our previous works [58,60]. For all experimental runs, a peristaltic pump (Reglo Digital, Ismatec, Glattbrugg, Switzerland) was used to continuously introduce liquid samples into the measurement cell at a constant flow rate of 100 µL/min. In between each experimental run, the measurement cell with the loaded chip was rinsed thoroughly by flowing into the cell a solution of 1 wt % sodium dodecyl sulfate (SDS) in water, water, and ethanol in succession for at least 10 min per step. The sensor chip itself was then removed and manually rinsed with the SDS solution, water and ethanol before air-drying with a gentle stream of nitrogen gas. Prior to each experimental run, the sensor chip was treated with oxygen plasma for 1 min (Harrick Plasma, Ithaca, NY, USA) and then loaded into the measurement cell. Of note, the oxygen plasma treatment results in the formation of a silica layer on the sensor surface and hence the coatings are referred to as silica coatings below. All LSPR data analysis was done using the Insplorer software package (Insplorion AB) with a time resolution of 1 Hz. The spectral resolution of the plasmon resonance as well as its centroid position was determined by high-order polynomial fitting [91].

### 2.6. Quartz Crystal Microbalance with Dissipation Monitoring

QCM-D measurements were performed using a Q-Sense E4 instrument (Q-Sense AB, Gothenburg, Sweden) with four parallel measurement chambers, each one of which contained a sputter-coated crystal with a 50-nm-thick silicon dioxide layer (QSX 303, Q-Sense AB) and a mass sensitivity constant of 17.7 ng/cm^2^·Hz. Before each measurement, the crystals were rinsed with water and ethanol, dried with a stream of nitrogen gas, and treated with oxygen plasma for 1 min (Harrick Plasma). Continuous flow conditions at a nominal rate of 0.1 mL/min were maintained in all measurements, as regulated by a peristaltic pump (Reglo Digital), and the temperature of the measurement cell was maintained at 25.0 ± 0.5 °C. Data were collected at the odd overtones (3 rd, 5 th, 7 th, 9 th and 11 th), and the normalized data at the 5th overtone are reported below. In between measurements, similar cleaning procedures were applied as described in Section 2.5.

## 3. Results and Discussion

### 3.1. Fabrication of SLB Coating

**Figure 1:**
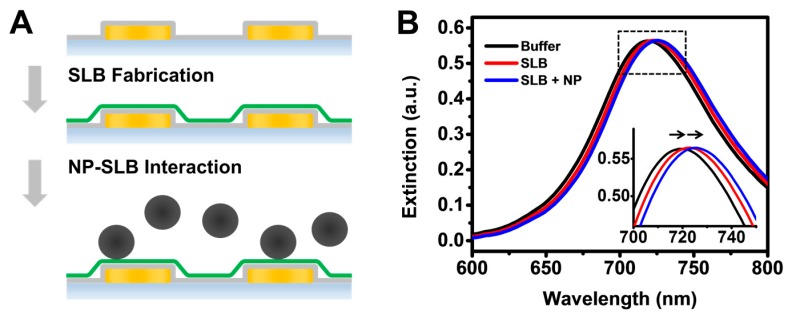
(**A**) Schematic illustration of supported lipid bilayer-coated (SLB-coated) nanodisk array for characterizing nanoparticle-SLB (NP-SLB) interactions; and (**B**) optical extinction spectra from localized surface plasmon resonance (LSPR) measurements show shifts in the λ_max_ position after SLB fabrication (denoted as SLB) and subsequent interaction with silica nanoparticles (denoted as SLB + NP). Inset shows an enlargement of the area bound by the dashed rectangle.

We utilized nanoplasmonic sensing platforms that consist of randomly distributed gold nanodisks on a glass surface, and the entire sensor surface was conformally coated with a 10-nm thick silica overlayer (Figure 1A). The silica layer was chosen because it facilitates the fabrication of SLB coatings via the well-established vesicle fusion approach using zwitterionic and positively-charged vesicles [92]. In this work, two fluid-phase lipid compositions were tested: zwitterionic 100 mol % 1,2-dioleoyl-*sn*-glycero-3-phosphocholine (DOPC) and positively-charged mixtures consisting of 70% DOPC mixed with 30% 1,2-dioleoyl-*sn*-glycero-3-ethylphosphocholine (DOPC:DOEPC 70:30). Accordingly, high-quality SLB coatings were fabricated via the vesicle fusion method using monodisperse vesicle suspensions of DOPC and DOPC:DOEPC 70:30 lipid compositions, with average sizes around 70 nm diameter as measured by dynamic light scattering (Figure S1, Supplementary Materials), and zeta potential of around −10 mV and +30 mV, respectively, as previously determined [93]. The LSPR extinction spectra before and after the SLB fabrication, as well as after the addition of SiNPs to the SLBs, are shown in Figure 1B. Due to adsorption of phospholipid molecules and/or SiNPs at the sensor surface, there was an accompanying increase in the local refractive index near the sensor surface and hence the wavelength of the LSPR peak position, λ_max_, increased (i.e., redshift) after SLB coating and subsequent introduction of SiNPs. While LSPR peak shifts provide information about changes in the local refractive index due to mass adsorption and rearrangement within the LSPR sensing volume, snapshot spectral data alone do not provide information on the kinetics of SLB fabrication process as well as nanoparticle–SLB interactions.

**Figure 2:**
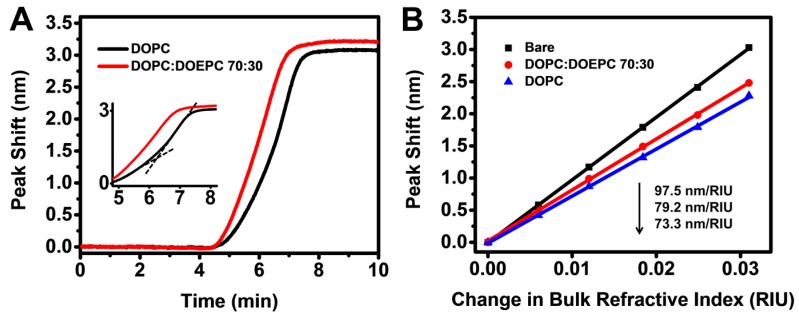
(**A**) Localized surface plasmon resonance (LSPR) peak shifts as a function of time during supported lipid bilayer (SLB) fabrication on silica-coated nanodisk arrays using zwitterionic DOPC and positively charged DOPC:DOEPC 70:30 vesicles. Inset shows the characteristic kink in the LSPR response, which is associated with the onset of vesicle rupture at a critical surface coverage of adsorbed vesicles; (**B**) LSPR peak shifts as a function of ΔRIU in glycerol/water mixtures. Linear fits show bulk refractive index sensitivities (nm/RIU) of bare silica-coated nanodisk arrays as well as silica-coated nanodisk arrays coated with DOPC and DOPC:DOEPC 70:30 SLBs.

To study the kinetics of the SLB fabrication process, we therefore tracked the LSPR peak shift, Δλ_max_, as a function of time. While redshifts were observed throughout the SLB fabrication process for both lipid compositions, there was a distinct variation in the kinetic profile between the two cases (Figure 2A). As part of the zwitterionic DOPC SLB formation process, a constant rate of increase in Δλ_max_ was initially observed before the rate accelerated suddenly, which was observed as a characteristic kink in the response signal implying SLB formation via vesicle rupture upon reaching a critical surface coverage of adsorbed vesicles [46,52]. In marked contrast, the kink was absent in the response signal during the formation of positively charged DOPC:DOEPC 70:30 SLBs. Rather, in this case, a monotonic increase in Δλ_max_ was observed, suggesting SLB formation via direct vesicle rupture [94]. For verification, QCM-D measurements were also performed in order to monitor SLB formation on silica-coated surfaces. The QCM-D signals showed that DOPC vesicles adsorbed until reaching saturation coverage at around −40 Hz before rupturing to form an SLB with a final frequency shift of around −25 Hz, while DOPC:DOEPC 70:30 vesicles directly ruptured to form an SLB, as indicated by a monotonic decrease in frequency which saturated at around −24 Hz (Figure S2). The QCM-D measurement values indicate SLB formation [93,95]. In addition to the difference in SLB formation kinetics, a higher final LSPR peak shift of ~3.21 nm was observed after the fabrication of the positively-charged DOPC:DOEPC 70:30 SLB, compared to a final peak shift of ~3.09 nm for the zwitterionic DOPC SLB. The observed trends agree well with our recent findings that, with increasing positive charge of the lipid composition, SLBs have a stronger net interaction with the underlying negatively charged silica surface, in effect stabilizing the SLB closer to the sensor surface and resulting in a higher LSPR peak shift due the measurement technique’s high surface sensitivity [94]. As the closely matching QCM-D frequency and dissipation shifts suggest that the two SLBs have similar qualities, it is noteworthy that the lower LSPR peak shift observed for the DOPC SLB is less likely to be due to a higher defect intensity since the QCM-D frequency shift is higher and dissipation shift is lower for this case, as compared to the DOPC:DOEPC 70:30 SLB.

To further characterize the optical sensing properties of the two SLB platforms, we compared the bulk refractive index sensitivities of the sensors before and after SLB fabrication via titration of glycerol-water mixtures (0–25 wt % glycerol) (Figure 2B). The bulk refractive index sensitivity, *S_B_*, reduced from 97.5 nm/RIU for the silica-coated nanodisk array before SLB fabrication to 79.2 nm/RIU and 73.3 nm/RIU after the fabrication of DOPC:DOEPC 70:30 and DOPC SLBs, respectively. Taking into account the ~5-nm thickness of the SLBs, the reduction in *S_B_* after the SLB coating supports that the sensing array has a short penetration depth, as further elaborated below. In addition, the DOPC:DOEPC 70:30 and DOPC SLBs have nearly identical optical properties (DOEPC is an ethylated analog of DOPC [96]), and hence the difference in *S_B_* can be attributed to the variation in SLB proximity to the sensor surface. Indeed, the lower *S_B_* value of the DOPC SLB is consistent with the fact that this SLB is stabilized farther away from the sensor surface as compared the DOPC:DOEPC 70:30 SLB. As a result, the probing volume sensitive to bulk refractive index changes is further away from the sensor surface in the DOPC SLB case and therefore in a region of lower field intensity.

To quantitatively characterize the surface sensitivity of the SLB-coated nanodisk arrays, we calculated the spatial proximity of the different SLBs to the sensor surface and the electromagnetic field decay length of the LSPR sensor based on the LSPR peak shift responses reported in Figure 2A. Generally, the distribution of the electromagnetic field intensity of the plasmon response as a function of the distance from the sensor surface is modeled as an exponential decay function with a characteristic decay length *L*. The effective refractive index, *n*_eff_, of the probing volume is denoted by

(1) $$n_{\mathrm{eff}}=\frac{1}{L}\int_{z=0}^{\infty }n\left(z\right)\exp\left(-z/L\right)dz$$

where *n*(*z*) is the refractive index at a distance *z* above the sensor surface. Here, *n*(*z*) = *n*_buffer_ for 0 < *z* < *d*, *n*(*z*) = *n*_SLB_ for *d* < *z* < *d* + *T*, and *n*(*z*) = *n*_buffer_ for *d* + *T* < *z* < infinity, where *d* is the distance from the SLB’s lower leaflet to the sensor surface and *T* is the optical thickness of the bilayer. In this case, *T* is taken to be 4.8 nm, considering a wet DOPC thickness of ~5.5 nm as reported in earlier works [97,98] and an expected interlayer water thickness of around 0.5 to 1.0 nm. The corresponding LSPR peak shift, Δλ_max_, which is induced by a change in the local refractive index, is given by

(2) $${\Delta \lambda }_{\max}=S\left(n_{\mathrm{eff}}-n_{\mathrm{buffer}}\right)$$

Substituting *n*_eff_ in Equation (2) with the integral of Equation (1), we can relate the final LSPR peak shift responses to the separation distances between the SLB and the sensor surface, *d*_DOPC_ and *d*_EPC_, for DOPC and DOPC:DOEPC 70:30 lipid compositions, respectively. However, numerical solutions cannot be obtained by this approach alone since the decay length, *L*, is unknown. To solve for *L*, it is necessary to consider variations in surface sensitivities for sensors coated with DOPC and DOPC:DOEPC 70:30 SLBs based on the bulk refractive index sensitivity values reported above. At this juncture, it is important to note that the calculated *L* represents the decay length of the bare sensor. In the presence of SLB coatings, the optical field will be distorted. The function describing the variation of *L* in the presence of SLB coatings is unknown and therefore has not been taken into account in our calculations. In any case, we expect *L* to be slightly lower in the presence of SLB coatings and consequently, the solution values reported herein represent reasonable but slightly over-estimated approximations. Typically, the surface sensitivity, *S_S_*, is related to the bulk refractive index sensitivity, *S_B_*, according to the following approximation [99]:

(3) $$S_{S}\approx S_{B}\exp\left(-2D/L\right)[1-\exp\left(-2t/L\right)],$$

where *t* is the thickness of the layer over which the refractive index change occurs, and *D* is the distance of the layer from the sensor surface. Upon formation of the SLB coating, the effective LSPR-probed sensing volume shifts away from the sensor surface. Assuming that this shift occurs over a vertical distance *D*, the surface sensitivity at the base of the sensing volume of an SLB-coated sensor can be simultaneously described by two equivalent forms of Equation (3). In the first form, the *S_B_* value is taken as the bulk refractive index sensitivity of the bare substrate in the absence of an SLB (i.e., 97.5 nm/RIU in the present experiments), in which case *D* needs to be solved for since we are measuring *S_S_* at a plane above the base of the bare sensing volume. In the second form, the *S_B_* value is taken as the bulk refractive sensitivity after SLB fabrication (i.e., 79.2 nm/RIU and 73.3 nm/RIU for DOPC:DOEPC 70:30 and DOPC SLBs, respectively). In this case, *D* will be zero since we are measuring *S_S_* at the base of the sensing volume after SLB fabrication. By approximating the difference between *D*_DOPC_ and *D*_EPC_ to be equal to the difference between *d*_DOPC_ and *d*_EPC_, numerical solutions to *d*_DOPC_, *d*_EPC_, *L*, *D*_DOPC_, and *D*_EPC_ can be simultaneously obtained and were calculated to be 1.04 nm, 0.51 nm, 13.45 nm, 1.93 nm and 1.40 nm, respectively (calculation details in Supplementary Materials). While we have previously shown that the nanoplasmonic sensing technique is capable of discerning sub-nanometer differences in SLB proximity to the sensor surface by considering only the final LSPR peak shifts after SLB formation [94], this is the first demonstration whereby absolute values of the separation distance between an SLB and the sensor surface were determined, by including bulk refractive index sensitivity measurements post-SLB coating as a refinement to the previous approach. In the previous work, the variation in bilayer-substrate separation distance for 100% PC versus 100% EPC SLBs on silica surfaces was estimated to be around 1.79 nm, and the absolute separate distances for these two cases were not explicitly determined. Based on the refinement used in this work, we not only determined the variation in separation distance (0.53 nm) for DOPC and DOPC:DOEPC 70:30 SLBs on silica surfaces, but also calculated the absolute separation distances (i.e., 1.93 and 1.40 nm for DOPC and DOPC:DOEPC 70:30 SLBs, respectively), which are in excellent agreement with the ranges quoted in literature reports [100,101,102]. The calculated *L* value of 13.45 nm also agrees well with previous reports, which suggest that the decay length of LSPR sensors typically lies in the range of 5–20 nm [103,104].

Finally, we characterized the sensor resolution before and after SLB coating, which was defined as the smallest change in the bulk refractive index that produced a detectable change in the output and was determined by dividing the standard deviation of the temporal noise of the sensor output by the bulk refractive index sensitivity [105]. For a bare sensor, the practical resolution was calculated to be 6.67 × 10^−5^ RIU. After the fabrication of DOPC and DOPC:DOEPC 70:30 SLB coatings, the values were 7.23 × 10^−5^ RIU and 7.88 × 10^−5^ RIU, respectively (details in Supplementary Materials). The similar resolutions (less than 20% variation) support that the LSPR sensor maintained its detection sensitivity after SLB coating in both cases.

### 3.2. Probing Nanoparticle Interactions with Supported Lipid Bilayers

**Figure 3:**
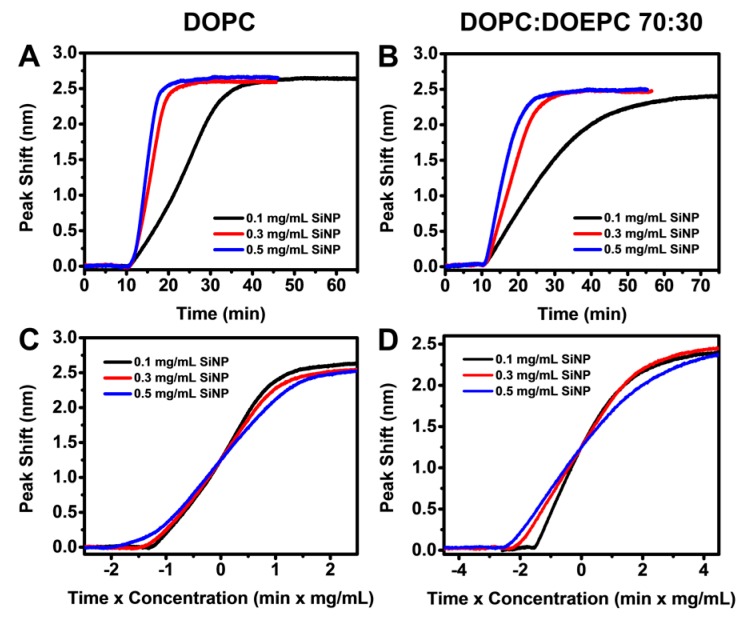
Localized surface plasmon resonance (LSPR) peak shifts as a function of time during the adsorption of silica nanoparticles (SiNP) onto: (**A**) DOPC; and (**B**) DOPC:DOEPC 70:30 SLBs. SiNPs were added at *t* = 10 min under continuous flow. The tested SiNP concentrations were 0.1, 0.3 and 0.5 mg/mL. Normalized responses from: (**C**) panel A; and (**D**) panel B scaled according to *ct* where *c* is the bulk SiNP concentration and *t* is time.

We next investigated the interaction of SiNPs with DOPC and DOPC:DOEPC 70:30 SLBs using highly monodisperse SiNPs measuring around 54 nm in diameter (Figure S3). When different bulk concentrations of SiNPs (0.1 to 0.5 mg/mL) were added to the DOPC SLB platform, the LSPR peak shift increased linearly due to SiNP adsorption onto the SLBs. As expected, the rate of change in the LSPR peak shift increased with bulk SiNP concentration, indicating quicker SiNP uptake up to saturation on account of diffusion-limited adsorption and higher bulk diffusion rates that are proportional to NP concentration (Figure 3A). It is also worthy to note that the total surface area of the nanoparticles is estimated to be at least 5 times higher than the surface area of SLB coated onto the sensor at a SiNP concentration of 0.1 mg/mL, and an order of magnitude higher at concentrations of 0.3 and 0.5 mg/mL. For all tested SiNP concentrations, the LSPR signals saturated at around 2.6 nm. An overall similar trend was observed when SiNPs were introduced to arrays coated with DOPC:DOEPC 70:30 SLBs (Figure 3B). However, compared to SiNP adsorption onto DOPC SLB, the LSPR signal reached saturation over a longer time scale for all tested concentrations on DOPC:DOEPC 70:30 SLBs. The final LSPR peak shifts were also slightly lower at around 2.5 nm. To account for concentration-dependence of the diffusion-limited adsorption, we reconstructed the LSPR peak shift responses as a function of *ct* where *c* is the bulk SiNP concentration and *t* is time. The *ct* curves for SiNP adsorption onto the DOPC SLB over the tested concentrations closely overlap, suggesting that the process follows the same pathway independent of NP concentration (Figure 3C). A similar trend was observed for SiNP adsorption onto DOPC:DOEPC 70:30 SLBs, although the *ct* curves extended over a wider range along the horizontal axis due to the relatively longer time needed for adsorption (Figure 3D). Taken together, the overlapping *ct* curves suggest that, on each SLB, the diffusion-limited rates of adsorption are equal and any variation in observed kinetics can therefore be attributed to differences in SLB + NP interactions.

**Figure 4:**
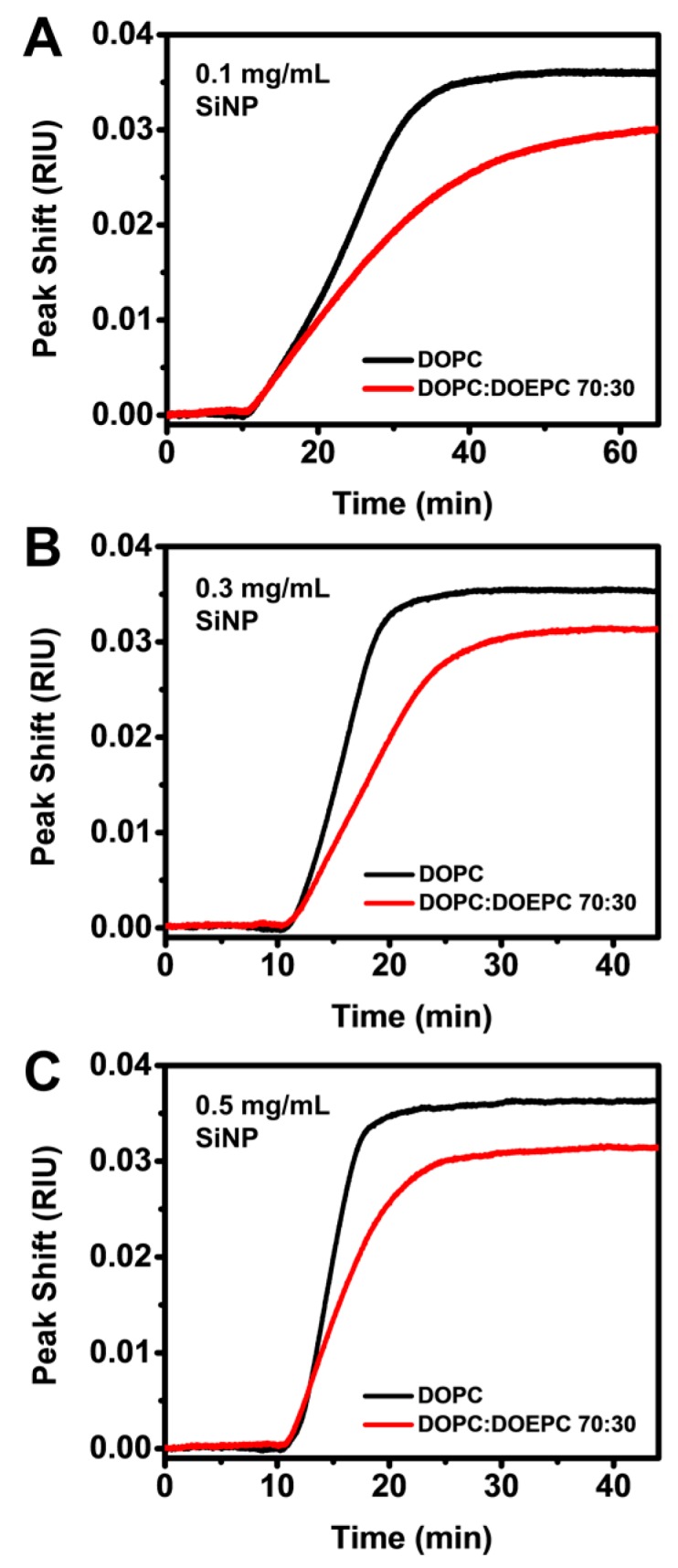
Normalized localized surface plasmon resonance (LSPR) peak shifts according to the experimentally determined bulk refractive index sensitivities of nanoplasmonic arrays with DOPC and DOPC:DOEPC 70:30 supported lipid bilayer (SLB) coatings, during the adsorption of silica nanoparticles (SiNPs) on the respective SLBs using bulk SiNP concentrations of: (**A**) 0.1 mg/mL; (**B**) 0.3 mg/mL; and (**C**) 0.5 mg/mL.

To further scrutinize the difference in interaction kinetics, we directly compared the LSPR responses due to SiNP accumulation on the two SLBs at each tested SiNP concentration. Considering the difference in bulk refractive index sensitivities between the SLB-coated sensors, the responses were normalized by dividing the LSPR peak shifts by the respective bulk refractive index sensitivities to directly compare the adsorption rates and saturation uptakes. While the raw LSPR responses showed a small difference in final peak shifts of around 0.1 nm, the normalized data revealed that SiNP uptake on the DOPC SLB was around 20% higher than on DOPC:DOEPC 70:30 SLB (Figure 4A–C). At a concentration of 0.1 mg/mL, the adsorption of SiNPs onto the two SLBs occurred at a similar rate in the initial stage but achieved quicker saturation uptake on DOPC SLB at around 40 min (Figure 4A). By contrast, the rate of SiNP uptake onto DOPC:DOEPC 70:30 SLBs gradually decreased approaching saturation and saturation uptake was achieved more than 20 min later. At 0.3 mg/mL SiNP concentration, a difference in the adsorption rate onto the two SLBs was also observed but it remained consistent throughout the process (Figure 4B). Saturation uptake was achieved in 25 min on the DOPC SLB and approximately 10 min later on the DOPC:DOEPC 70:30 SLB. A similar trend was observed at the highest tested concentration but the time difference to reach saturation uptake was further reduced to around 5 min (Figure 4C).

The trends observed in the normalized data indicate higher rates of SiNP adsorption and saturation uptakes on DOPC SLBs compared to DOPC:DOEPC 70:30 SLBs. This observation is surprising because it is expected that nanoparticle adsorption would be diffusion-limited [106] in both cases, and negatively-charged SiNPs have greater electrostatic attraction to the positively charged DOPC:DOEPC SLB. To explain the deviation between the observed and expected results, the findings support that there is a strong electrostatic interaction between negatively-charged SiNPs and positively-charged DOPC:DOEPC 70:30 SLBs, inducing the detachment of likely DOEPC lipids from the SLB followed by their subsequent adherence to the SiNP surface. The extraction of positively charged lipids from mixed membrane assemblies by adsorbing negatively charged nanoparticles (gold nanoparticles and quantum dots) has been previously observed by Xiao et al., and it was described that lipid transfer from the membrane assembly to the nanoparticles led to domain-specific membrane disruption [107]. Interestingly, they reported that maximum amount of disruption occurred at around 40% positively charged lipid fractions, which is close to our case of 30% positively charged DOECP lipids and supports our observations. Likewise, lipid mass transfer from an SLB to semihydrophobic nanoparticles occurred during the adsorption of the nanoparticles followed by the nanoparticle extraction of surrounding lipids leading to regions with low lipid density [108]. Investigations by Bailey et al. revealed that the tendency for lipid bilayers to be extracted by nanoparticles depends on the particle–bilayer adhesion energy, bilayer bending energy and interfacial energy at bilayer defects [83]. As a result, even though larger particles have lower surface energy, there is a higher propensity for lipid extraction since the bilayer can overcome the lower bending energy to adhere to the particle surface with lesser curvature.

In general, in an SLB + NP system, a mass increase on top of the SLB can be attributed to the accumulation of nanoparticles onto the SLB as well as nanoparticle deformation, if the nanoparticle is deformable. In our case, the diffusion-limited rate of SiNP adsorption onto the two SLBs is equal and the SiNPs are rigid and do not deform during the adsorption process. Therefore, if the only contribution to the LSPR signals is a mass increase on top of the SLB, higher SiNP adsorption rates and saturation uptakes would have been observed on positively charged DOPC:DOEPC 70:30 SLBs (and equivalent to those observed in the DOPC SLB case) since there are no competing contributions to the LSPR signal aside from the difference in SLB + NP interaction. Conversely, this means that changes to the SLB properties need to be taken into consideration to explain the deviation as suggested above. During the adsorption of SiNPs onto the SLBs, any lipid mass transfer from the SLB to the SiNP surface would lead to a lower lipid mass density and hence lower effective refractive index on the sensor surface, resulting in a decreased net LSPR response due to the competing influences of SiNP adsorption and lipid transfer from the sensor surface to the SiNP. As our nanoplasmonic sensing platform represents an ensemble measurement technique, the LSPR response takes into account the replacement of the extracted lipids by lateral diffusion of lipids from neighboring regions at the sensor surface (i.e., self-healing), since the overall effective refractive index on the sensor surface will be lower even after self-healing. In this case, stronger electrostatic interactions between negatively charged SiNPs and a positively charged DOPC:DOEPC 70:30 SLB led to the extraction of positively charged lipid domains to the SiNP surface when the SiNP approaches the SLB, in a similar manner to that observed by Xiao et al. between negatively charged nanoparticles and positively charged SLBs. As a result, lower apparent adsorption rates and saturation uptakes were observed on DOPC:DOEPC 70:30 SLBs based on the LSPR peak shifts.

To support the aforementioned description of the scenario, QCM-D measurements were performed and the frequency shifts showed closely similar saturation uptakes and adsorption kinetics, accompanied by minor variations in the dissipation shifts (Figure S4). This implies that within the appreciably larger probing volume of the QCM-D measurements (up to ~100–150 nm from the sensor surface) that encompasses the length scale of the nanoparticles, the same degree of overall mass accumulation occurred on the sensors coated with the two SLBs. Indeed, even if lipid mass was transferred from the sensor surface to the SiNP surface for the DOPC:DOEPC 70:30 SLB case, all the lipid mass would remain within the QCM-D probing volume and hence no difference in the QCM-D measurement responses would occur, which is indeed the case we observe here. This finding also supports that the lipid mass removed from the sensor surface remains bound to the SiNP surface (and hence remains within the QCM-D probing volume), although we cannot fully exclude that some lipid mass is possibly solubilized and released from the system. We can therefore infer that the variations observed in the LSPR responses for SiNP adsorption onto the two SLBs are due to a difference in the resulting spatial distribution of the lipid mass close to the sensor surface, and hence our proposed explanation is fully consistent across the LSPR and QCM-D measurements. As such, the data indicate that SiNPs adsorb onto both DOPC and DOPC:DOEPC 70:30 SLBs, and have stronger interactions with DOPC:DOEPC 70:30 SLBs causing membrane disruption and lipid detachment from the sensor surface. Of note, the removal of the lipids from the sensor surface would slightly increase the surface sensitivity of the sensor and compensating for this effect would further support this scenario.

**Figure 5:**
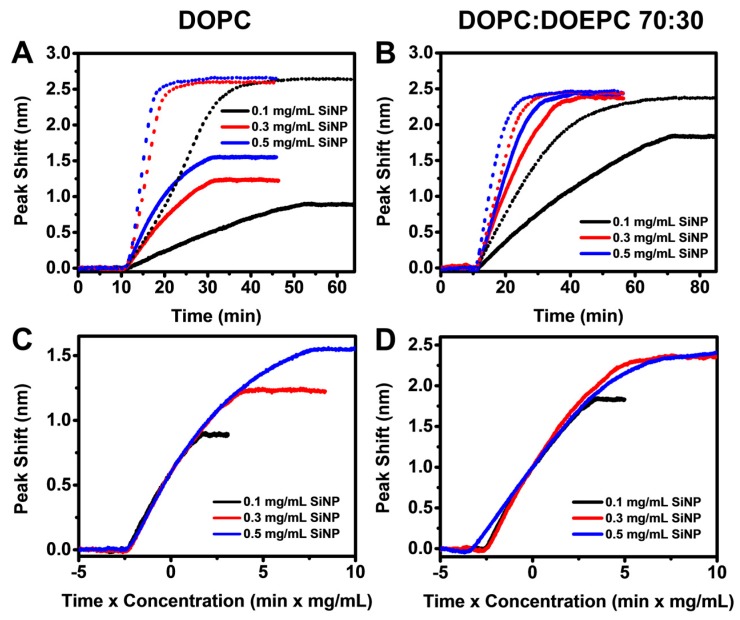
Localized surface plasmon resonance (LSPR) peak shifts as a function of time during the adsorption of bovine serum albumin (BSA)-coated silica nanoparticles (SiNP) at concentrations of 0.1, 0.3 and 0.5 mg/mL on: (**A**) DOPC; and (**B**) DOPC:DOEPC 70:30 SLBs. BSA-coated SiNPs were added at *t* = 10 min. Responses during the adsorption of bare SiNP are also included as dotted lines for comparison. Normalized responses from: (**C**) panel A; and (**D**) panel B scaled according to *ct* where *c* is the bulk SiNP concentration and *t* is time.

Motivated by these findings, we next explored surface functionalization schemes in order to inhibit nanoparticle-induced membrane interactions, specifically precoating SiNPs with bovine serum albumin (BSA) through noncovalent adsorption [86,109,110]. Indeed, recent findings point to the significant role of protein coatings in diminishing electrostatic interactions between charge-bearing nanoparticles and cell membranes [111]. In particular, it was found that the coating of SiNPs with BSA resulted in an increase in the zeta potential of around +5 mV at physiological pH [112,113]. Following this approach, we precoated the SiNPs with BSA in order to modulate SiNP adsorption onto the SLBs. The BSA-coated SiNPs measured around 79 nm in diameter, which further supports BSA adsorption (Figure S5). When added to DOPC SLBs, BSA-coated SiNPs exhibited significantly lower adsorption rates and saturation uptakes compared to bare SiNPs (Figure 5A). This is due to a lower particle–bilayer interaction energy of the BSA-coated SiNPs leading to reduced nonspecific interactions with the SLB [114] as well as increased steric repulsions between BSA-coated SiNPs and the SLB [86].

While the adsorption of bare SiNPs onto DOPC SLBs resulted in peak shifts of around 2.7 nm, the adsorption of BSA-coated SiNPs resulted in peak shifts of around 0.9 nm to 1.6 nm, depending on the nanoparticle concentration. These observations support that the interaction between SiNPs and DOPC SLBs can be inhibited by introducing a noncovalent protein coating around the SiNP. We then adsorbed BSA-coated SiNPs onto DOPC:DOEPC 70:30 SLBs. Compared to SiNP adsorption on DOPC SLBs, the difference in adsorption kinetics was less significant for all tested concentrations and the final saturation peak shifts were closely similar to the case of bare SiNPs at concentrations of 0.3 and 0.5 mg/mL, indicating comparable uptakes (Figure 5B). This finding is reasonable considering a stronger electrostatic interaction between negatively-charged BSA-coated SiNPs (considering BSA is negatively charged at physiological pH [115]) and positively-charged DOPC:DOEPC 70:30 SLBs, resulting in higher saturation peak shifts for all tested SiNP concentrations compared to the case of the zwitterionic DOPC SLB. It is also important to note that, for BSA-coated SiNPs, QCM-D measurements revealed variations in the frequency shifts that match the LSPR measurements for both SLB cases, suggesting a difference in the overall extent of mass uptake instead of mass distribution as in the case of bare SiNPs (Figure S6). Slightly lower final dissipation shifts observed on DOPC:DOEPC 70:30 SLBs also indicate a more rigid assembly of BSA-coated SiNPs.

The *ct* curves for the adsorption of BSA-coated SiNPs onto DOPC SLBs perfectly overlap, with no variation in the gradients at any stage (Figure 5C). This signifies that the adsorption of BSA-coated SiNPs at the tested concentrations follows the same pathway. Considering the relative low surface coverages of BSA-coated SiNPs on the SLBs throughout the adsorption process, this observation is consistent with expectations. On the other hand, the *ct* curves for the adsorption BSA-coated SiNPs onto DOPC:DOEPC 70:30 SLBs are not perfectly overlapping (Figure 5D). However, they more closely overlap compared to the case of bare SiNPs on DOPC:DOEPC 70:30 SLBs. This suggests that, unlike bare SiNPs, the adsorption of BSA-coated SiNPs onto DOPC:DOEPC 70:30 SLBs did not involve lipid transfer from the SLB to the SiNP surface. Instead, it follows a simple adsorption pathway. In other words, the BSA coating of SiNPs prevents SiNP extraction of lipids from the DOCP:DOEPC 70:30 SLB upon adsorption, and verified the mechanistic explanation proposed above as well as support that BSA coatings can inhibit SiNP adsorption onto both zwitterionic and positively charged lipid membranes.

## 4. Conclusions

We have fabricated SLB-coated nanoplasmonic sensing platforms functionalized with zwitterionic DOPC and positively-charged DOPC:DOEPC 70:30 SLBs on silica-coated gold nanodisks via the vesicle fusion method. Depending on the lipid composition, the bulk refractive index sensitivities of the sensing platform varied due to different SLB proximities to the underlying silica surface. Taking into account the correlation between bulk refractive index sensitivity and SLB proximity to the surface, we demonstrated for the first time a quantitative approach to determine the absolute values of the respective SLB separation distances from the surface based on LSPR measurements. These values were simultaneously obtained along with the electromagnetic field decay length, or penetration depth, of the sensor as well as the shifts in the sensing volume away from the sensor surface as a result of SLB fabrication. Of note, we found that the sensing volume shifted at distances much smaller than the SLB thicknesses, suggesting that the sensor was sensitive to changes in refractive index not only at the SLB–bulk interface but dynamic responses occurring with SLBs (e.g., lipid transfer). Obtaining such information was critical for an accurate interpretation of LSPR responses arising from nanoparticle–SLB interactions. In particular, we found that the interaction of negatively-charged bare SiNPs with zwitterionic DOPC SLBs led to simple adsorption while a stronger nanoparticle–membrane interaction with positively-charged DOPC:DOEPC 70:30 SLB led to lipid transfer from the SLB to the SiNP surface, resulting in lower net LSPR peak shifts. By contrast, the interaction of negatively-charged BSA-coated SiNPs with both SLBs resulted in simple adsorption with higher uptake on the positively-charged SLB, resulting in higher LSPR peak shifts. Taken together, the results in this work demonstrate that SLB-coated nanoplasmonic sensing arrays represent an ideal platform for investigating nanoparticle–membrane interactions due to an excellent agreement between its narrow penetration depth and the position of the nanoparticle–SLB interface. This match not only enabled the detection of subtle differences in the adsorption kinetics but also structural transformations at the SLB–NP interface arising from differences in nanoparticle–SLB interactions. Furthermore, as demonstrated in this work, our platform enables investigations involving functionalized nanoparticles. At the same time, the coated SLBs can be incorporated with membrane receptor proteins, allowing investigations into specific interactions between nanoparticles and SLBs. With a simple fabrication strategy and established methods to quantitatively characterize the platform and interpret the LSPR measurement responses, we expect the platform to be conveniently adopted for the investigation of other nanoparticle–membrane interaction systems as well as extended to a wide range of biointerfacial science applications.

## Supplementary Materials

The following are available online at http://www.mdpi.com/1424-8220/17/7/1484/s1. Calculation details and Figures S1–S6.
