# Supplementary material for: Probing the Interaction of Dielectric Nanoparticles with Supported Lipid Membrane Coatings on Nanoplasmonic Arrays
Source: Sensors (Basel). 2017 Jun 23;17(7):1484. doi: 10.3390/s17071484 (PMC5539686; doi:10.3390/s17071484)
Supplement: Supplementary file 1 [file sensors-17-01484-s001.pdf]

Supplementary Data

Probing the Interaction of Dielectric Nanoparticles with Supported Lipid Membrane Coatings on Nanoplasmonic Arrays

Abdul Rahim Ferhan [1,†], Gamaliel Junren Ma [1,†], Joshua A. Jackman [1], Tun Naw Sut [1], Jae Hyeon Park [1] and Nam-Joon Cho [1,2,*]

[1] School of Materials Science and Engineering and Centre for Biomimetic Sensor Science, Nanyang Technological University, 50 Nanyang Drive, 637553, Singapore; ferhan@ntu.edu.sg (A.R.F.); gma001@e.ntu.edu.sg (G.J.M.); jjackman@ntu.edu.sg (J.A.J.); suttun001@e.ntu.edu.sg (T.N.S.); park0005@e.ntu.edu.sg (J.H.P.)

[2] School of Chemical and Biomedical Engineering, Nanyang Technological University, 62 Nanyang Drive, 637459, Singapore

† These authors contributed equally to this work.

* Correspondence: njcho@ntu.edu.sg; Tel.: +65-6790-4925

Determination of SLB-Sensor Surface Separation Distances and LSPR Electromagnetic Field Decay Length

The separation distances between the SLBs and the sensor surface as well as the electromagnetic field decay length of the sensor were determined by taking into consideration both the difference in LSPR peak shifts as well as the difference in sensor bulk refractive index sensitivities after SLB fabrication.

The effective refractive index of the LSPR-probed sensing volume is denoted by

$$n_{\mathrm{eff}} = \frac{1}{L}\int_{z=0}^{\infty} n(z)\exp(-z/L)\,dz, \tag{1}$$

where $n(z)$ is the refractive index at a distance z above the sensor surface and L is the decay length of the sensor. Here, $n(z) = n_{\mathrm{buffer}}$ for $0 < z < d$, $n(z) = n_{\mathrm{SLB}}$ for $d < z < d + T$, and $n(z) = n_{\mathrm{buffer}}$ for $d + T < z <$ infinity, where d is the distance from the SLB's lower leaflet to the sensor surface and T is the dry bilayer thickness. Integration of Eq. 1 gives

$$n_{\mathrm{eff}} = \frac{n_{\mathrm{buffer}}}{L}\int_{z=0}^{d} \mathrm{e}^{(-z/L)} + \frac{n_{\mathrm{SLB}}}{L}\int_{z=d}^{d+T} \mathrm{e}^{(-z/L)} + \frac{n_{\mathrm{buffer}}}{L}\int_{z=d+T}^{\infty} \mathrm{e}^{(-z/L)}$$

$$= n_{\mathrm{buffer}}\left[1 - \mathrm{e}^{(-d/L)} + \mathrm{e}^{(-(d+T)/L)}\right] + n_{\mathrm{SLB}}\left[\mathrm{e}^{(-d/L)} - \mathrm{e}^{(-(d+T)/L)}\right], \tag{2}$$

The LSPR peak shift, $\Delta\lambda_{\mathrm{max}}$, induced by the change in the local refractive index is given by

$$\Delta\lambda_{\mathrm{max}} = S(n_{\mathrm{eff}} - n_{\mathrm{buffer}}), \tag{3}$$

Substituting Eq. 2 into Eq. 3, we obtain the following equation

$$\Delta\lambda_{\mathrm{max}} = S\{n_{\mathrm{buffer}}\left[\mathrm{e}^{(-(d+T)/L)} - \mathrm{e}^{(-d/L)}\right] + n_{\mathrm{SLB}}\left[\mathrm{e}^{(-d/L)} - \mathrm{e}^{(-(d+T)/L)}\right]\}, \tag{4}$$

In this case, S refers to the bulk refractive index sensitivity of the bare sensor, which was experimentally determined to be 97.5 nm/RIU. The refractive index of the buffer, n_{buffer}, was measured using an Abbe refractometer and was determined to be 1.336. The refractive index of the SLB, n_{SLB}, is taken to be 1.45, as reported previously [1]. The dry thickness of the SLB, T, is assumed to be 4.8 nm based on previous works that report the wet thickness of DOPC lipid bilayers to be around 5.5 nm [2, 3] and taking into consideration an expected interlayer water thickness of ~0.5 to 1.0 nm. Substituting these values, along with the LSPR peak shift responses after the fabrication of the SLBs,

the correlation between the LSPR response, $\Delta\lambda_{max}$, the separation distance between the DOPC:DOEPC 70:30 SLB and sensor surface, d_{EPC}, as well as the decay length, L, becomes

$$3.21 = 97.5\{1.336[e^{(-(d_{EPC}+4.8)/L)} - e^{(-d_{EPC}/L)}] + 1.450[e^{(-d_{EPC}/L)} - e^{(-(d_{EPC}+4.8)/L)}]\}, \quad (5)$$

The corresponding equation accounting for the separation distance between the DOPC SLB and sensor surface, d_{PC}, becomes

$$3.09 = 97.5\{1.336[e^{(-(d_{PC}+4.8)/L)} - e^{(-d_{PC}/L)}] + 1.450[e^{(-d_{PC}/L)} - e^{(-(d_{PC}+4.8)/L)}]\}, \quad (6)$$

Since numerical solutions to the three unknown variables d_{EPC}, d_{PC}, and L cannot be obtained using only Eqs. 5 & 6, it is necessary to take into account the difference in refractive index sensitivities. Generally, the surface sensitivity, S_S, at a certain distance above the sensor is related to the bulk refractive index sensitivity, S_B, according to following approximation [4]

$$S_S \approx S_B \exp(-2D/L)[1 - \exp(-2t/L)], \quad (7)$$

where t is the thickness of the layer over which the refractive index change occurs (*i.e.*, thickness of the LSPR-probed sensing volume), and D is the distance of the layer from the sensor surface. After fabrication of the SLB coating, the base of the LSPR-probed sensing volume, which corresponds to the sensing interface with the bulk environment, is expected to shift away from the sensor surface. Assuming that it shifts by a distance D, we can relate the bulk refractive index sensitivities before and after SLB coating by equating the surface sensitivity at the base of the LSPR-probed sensing volume after SLB coating to the surface sensitivity at distance D in the absence of SLB (*i.e.*, bare sensor surface). Specifically, the surface sensitivity at the base of the LSPR-probed sensing volume after the fabrication of DOPC:DOEPC 70:30 SLB, $S_{S,EPC}$, can be described as follows

$$S_{S,EPC} \approx S_{B,bare} \exp(-2D_{EPC}/L)[1 - \exp(-2t/L)] \approx S_{B,EPC}[1 - \exp(-2t/L)], \quad (8)$$

where D_{EPC} is the vertical distance over which the LSPR-probed sensing volume is moved away from the sensor surface due to the fabrication of DOPC:DOEPC 70:30 SLB. Substituting the experimentally determined bulk refractive index sensitivity values, the correlation between bulk refractive index sensitivities before and after SLB fabrication becomes

$$97.5 \exp(-2D_{EPC}/L) \approx 79.2, \quad (9)$$

For the case of the DOPC SLB, which is further away from the sensor surface, the base of the sensing layer will shift by a distance greater than D_{EPC}. We approximate the additional distance to be equal to the difference in separation distance between the SLBs and the sensor surface. Hence, for the DOPC SLB case, the correlation between bulk refractive index sensitivities before and after SLB fabrication will simplify to give

$$97.5 \exp(-2D_{PC}/L) \approx 73.3, \quad (10)$$

and

$$D_{PC} - D_{EPC} \approx d_{PC} - d_{EPC}, \quad (11)$$

By solving Eqs. 5, 6, 9, 10, & 11 simultaneously, numerical solutions to d_{EPC}, d_{PC}, L, D_{EPC}, and D_{PC} were calculated to be 0.51 nm, 1.04 nm, 13.45 nm, 1.40 nm, and 1.93 nm, respectively.

Determination of Practical Sensor Resolution

Following the approach established by Homola [5], the resolution of the sensor is defined as

$$r_{RI} = \frac{\sigma_{SO}}{S}, \quad (12)$$

where σ_{SO} is the standard deviation of noise of the sensor output and S is the bulk refractive index sensitivity. The standard deviation of noise obtained from the baseline during the refractive index sensitivity measurements before SLB coating was found to be at 0.00650 nm. After coating, the values were 0.00530 nm and 0.00495 nm, for the DOPC and DOPC:DOEPC 70:30 SLBs, respectively. By substituting the values of σ_{SO} = 0.00650 nm and S = 97.5 nm/RIU into Eq. 12 for bare substrate, a

resolution of 6.67 x 10^{-5} RIU was obtained. For the DOPC SLB, the substituted values were σ_{SO} = 0.00530 nm and *S* = 73.3 nm/RIU, giving a resolution of 7.23 x 10^{-5} RIU. For the DOEPC:DOPC 70:30 SLB, the substituted values were σ_{SO} = 0.00624 nm and *S* = 79.2 nm/RIU, giving a resolution of 7.88 x 10^{-5} RIU.

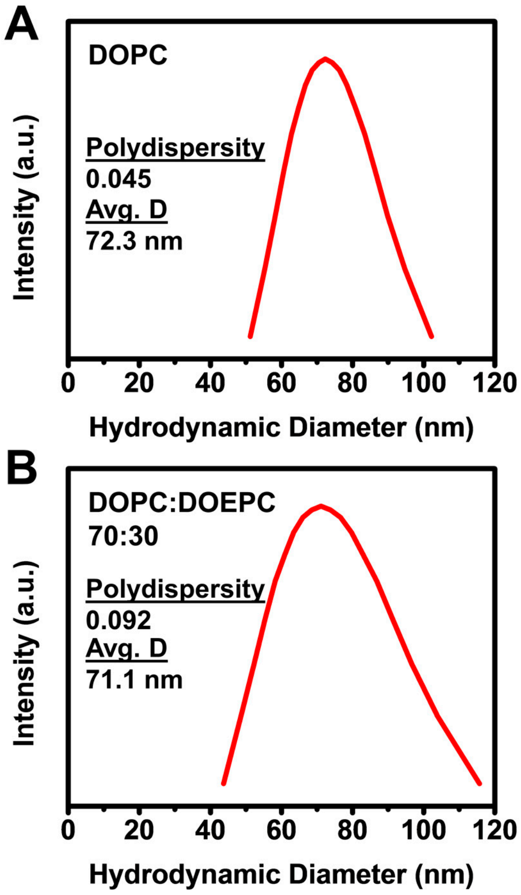

Figure S1. Lognormal size distributions of the (**A**) DOPC and (**B**) DOPC:DOEPC 70:30 vesicle populations plotted as intensity versus hydrodynamic diameter. The highest intensity value corresponds to the intensity-weighted average hydrodynamic diameter. The polydispersity values are also included.

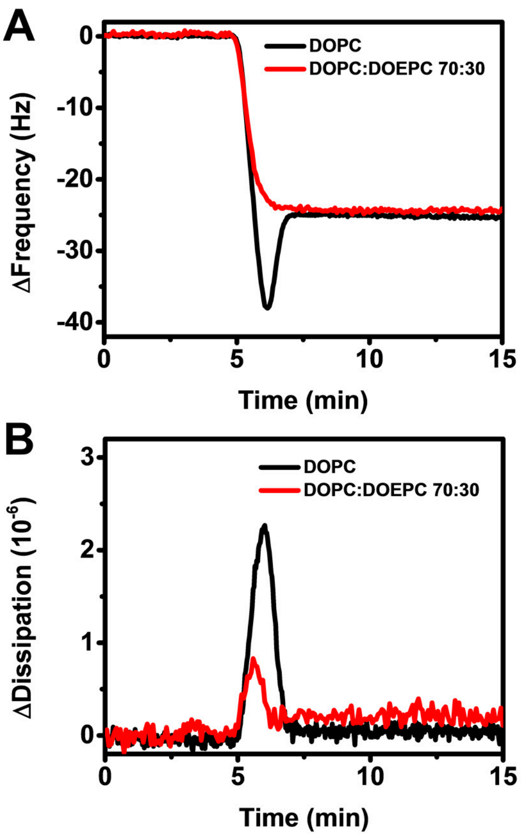

Figure S2. QCM-D (**A**) frequency and (**B**) dissipation shifts recorded as a function of time during the fabrication of SLB on the silica-coated nanodisk arrays using zwitterionic DOPC and positively charged DOPC:DOEPC 70:30 vesicles.

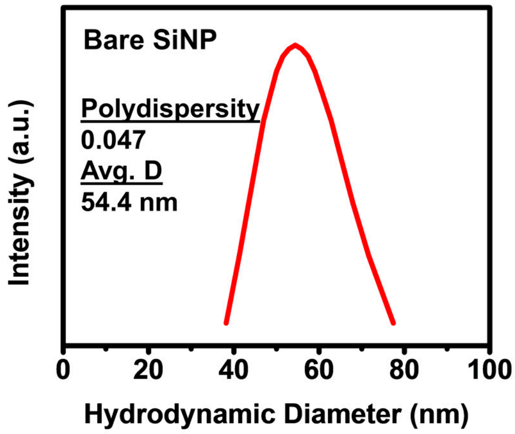

Figure S3. Lognormal size distribution of bare silica nanoparticle (SiNP) population plotted as intensity versus hydrodynamic diameter. The highest intensity value corresponds to the intensity-weighted average hydrodynamic diameter, which is indicated along with the polydispersity value.

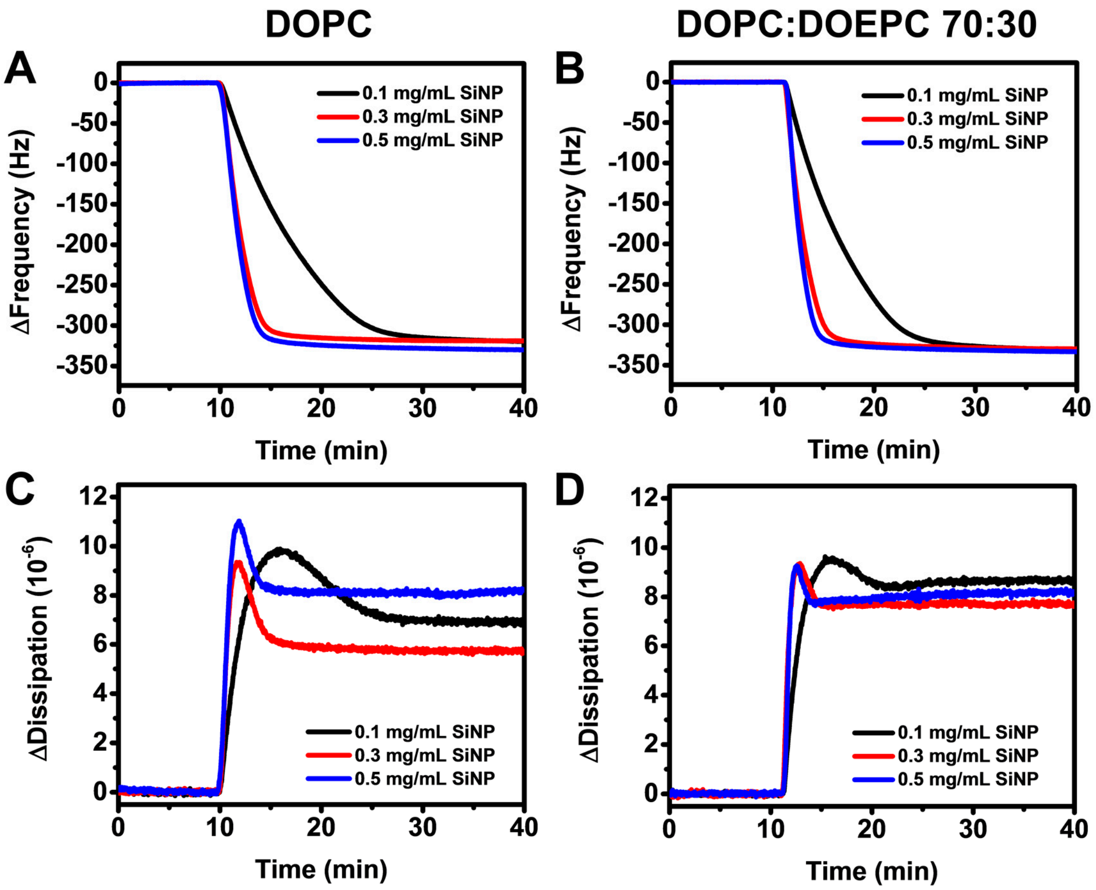

Figure S4. QCM-D frequency shifts as a function of time during the adsorption of silica nanoparticles (SiNP) at concentrations of 0.1, 0.3 and 0.5 mg/mL on (**A**) DOPC and (**B**) DOPC:DOEPC 70:30 SLBs. Corresponding QCM-D dissipation shifts to the frequency shifts in (**C**) panel A and (**D**) panel B.

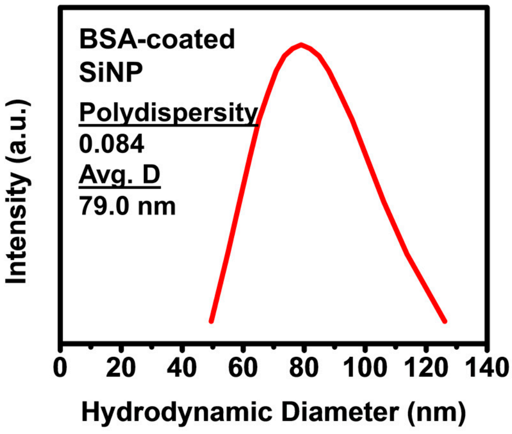

Figure S5. Lognormal size distribution of BSA-coated silica nanoparticle (SiNP) population plotted as intensity versus hydrodynamic diameter. The highest intensity value corresponds to the intensity-weighted average hydrodynamic diameter, which is indicated along with the polydispersity value.

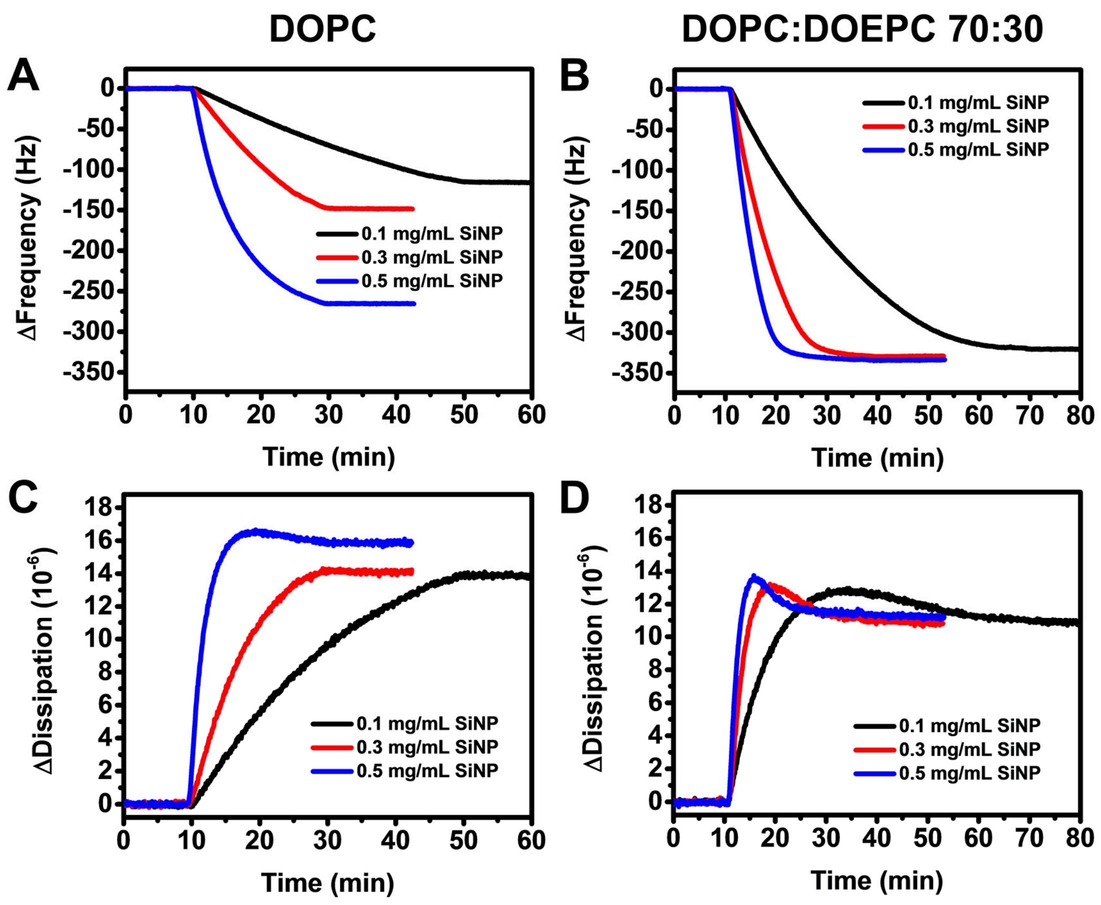

Figure S6. QCM-D frequency shifts as a function of time during the adsorption of BSA-coated silica nanoparticles (SiNP) at concentrations of 0.1, 0.3 and 0.5 mg/mL on (**A**) DOPC and (**B**) DOPC:DOEPC 70:30 SLBs. Corresponding QCM-D dissipation shifts to the frequency shifts in (**C**) panel A and (**D**) panel B.

References

1. Cho, N.-J.; Wang, G.; Edvardsson, M.; Glenn, J. S.; Hook, F.; Frank, C. W. Alpha-Helical Peptide-Induced Vesicle Rupture Revealing New Insight into the Vesicle Fusion Process As Monitored in Situ by Quartz Crystal Microbalance-Dissipation and Reflectometry. *Anal. Chem.* **2009,** *81*, 4752-4761.

2. Leonenko, Z. V.; Finot, E.; Ma, H.; Dahms, T. E. S.; Cramb, D. T. Investigation of Temperature-Induced Phase Transitions in DOPC and DPPC Phospholipid Bilayers Using Temperature-Controlled Scanning Force Microscopy. *Biophys. J.* **2004,** *86*, 3783-3793.

3. Morigaki, K.; Schönherr, H.; Frank, C. W.; Knoll, W. Photolithographic Polymerization of Diacetylene-Containing Phospholipid Bilayers Studied by Multimode Atomic Force Microscopy. *Langmuir* **2003,** *19*, 6994-7002.

4. Jackman, J. A.; Ferhan, A. R.; Cho, N.-J. Nanoplasmonic sensors for biointerfacial science. *Chem. Soc. Rev.* **2017**.

5. Homola, J. Surface Plasmon Resonance Sensors for Detection of Chemical and Biological Species. *Chem. Rev.* **2008,** *108*, 462-493.
